# Supplementary material for: Investigation of Electrical Transitions in the First Steps of Spark Plasma Sintering: Effects of Pre-Oxidation and Mechanical Loading within Copper Granular Media
Source: Materials (Basel). 2022 Jun 9;15(12):4096. doi: 10.3390/ma15124096 (PMC9227404; doi:10.3390/ma15124096)
Supplement: Supplementary file 1 [file materials-15-04096-s001.zip › materials-1751666-supplementary.pdf]

Supplementary Material to the paper Investigation of electrical transitions in the first steps of spark plasma sintering: effects of pre-oxidation and mechanical loading within copper granular media

Anis Aliouat [1], Guy Antou [1], Vincent Rat [1], Nicolas Pradeilles [1], Pierre -Marie Geffroy [1], Alexandre Maître [1]

[1] Institute for Research on Ceramics (IRCER), UMR CNRS 7315, Univ. Limoges, F-87068 Limoges, FRANCE

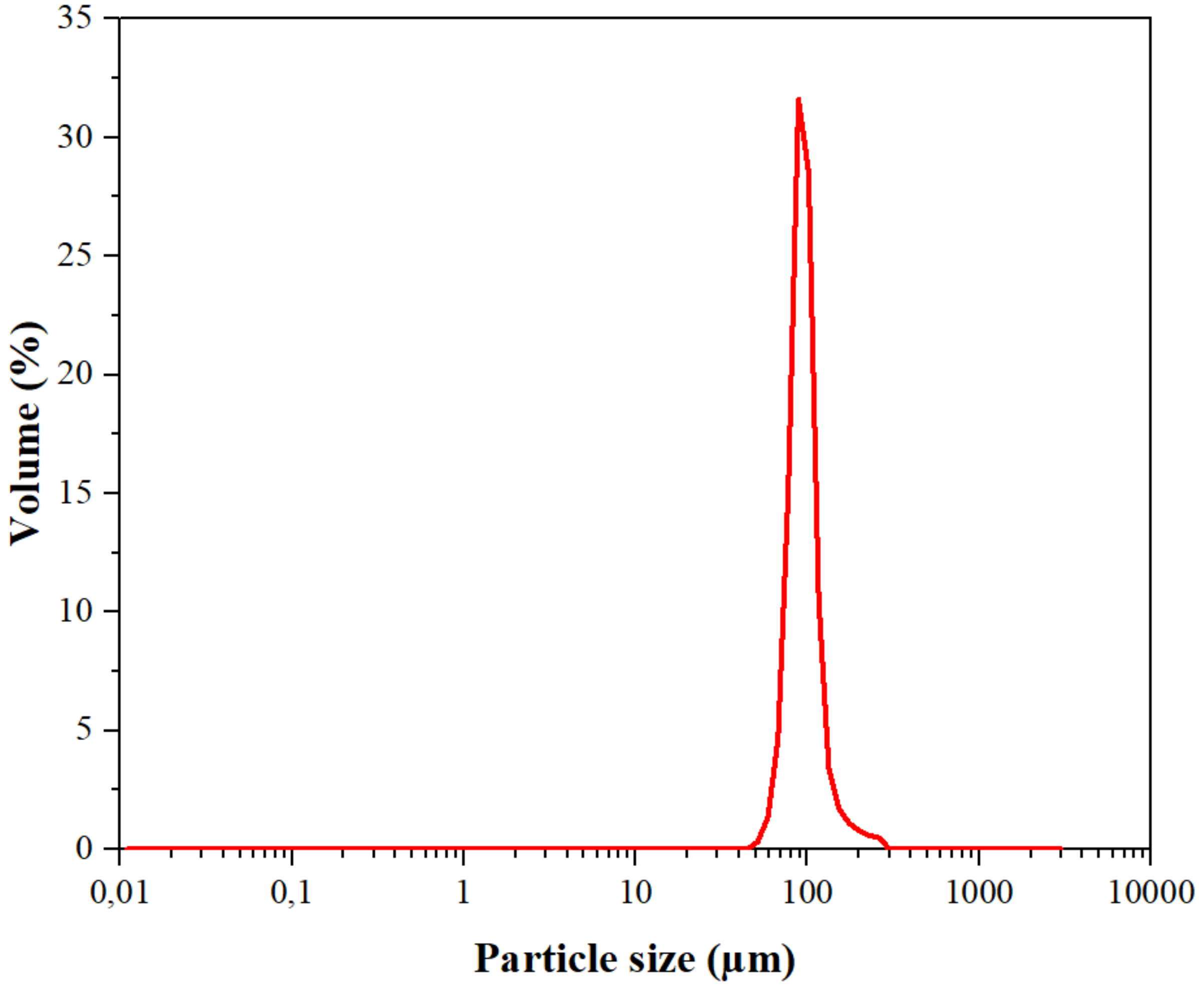

Figure S1. Particle size distribution of raw copper powder.

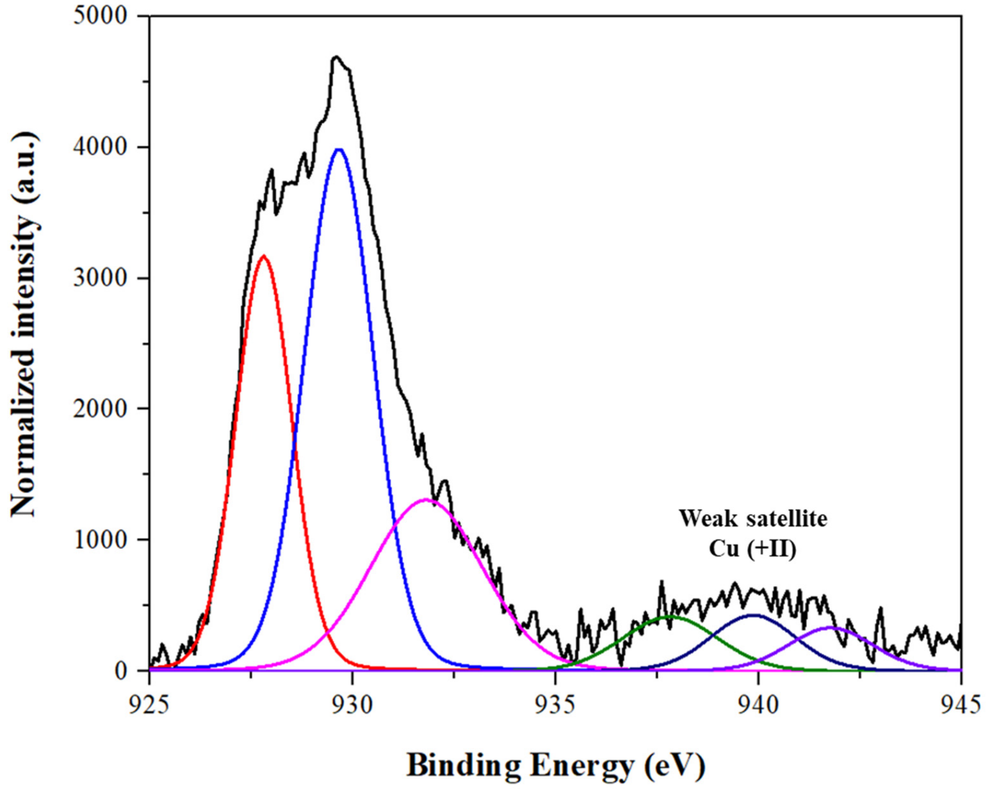

Figure S2. XPS spectrum of reduced copper powder at 400 °C.

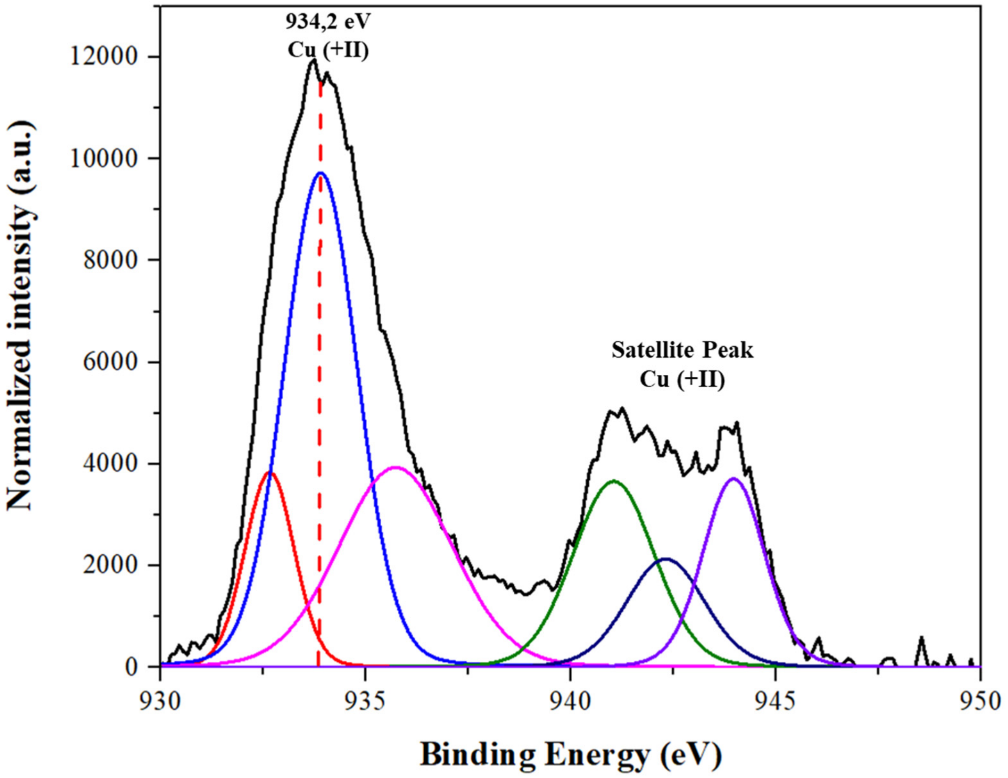

Figure S3. XPS spectrum of oxidized copper powder at 280 °C.

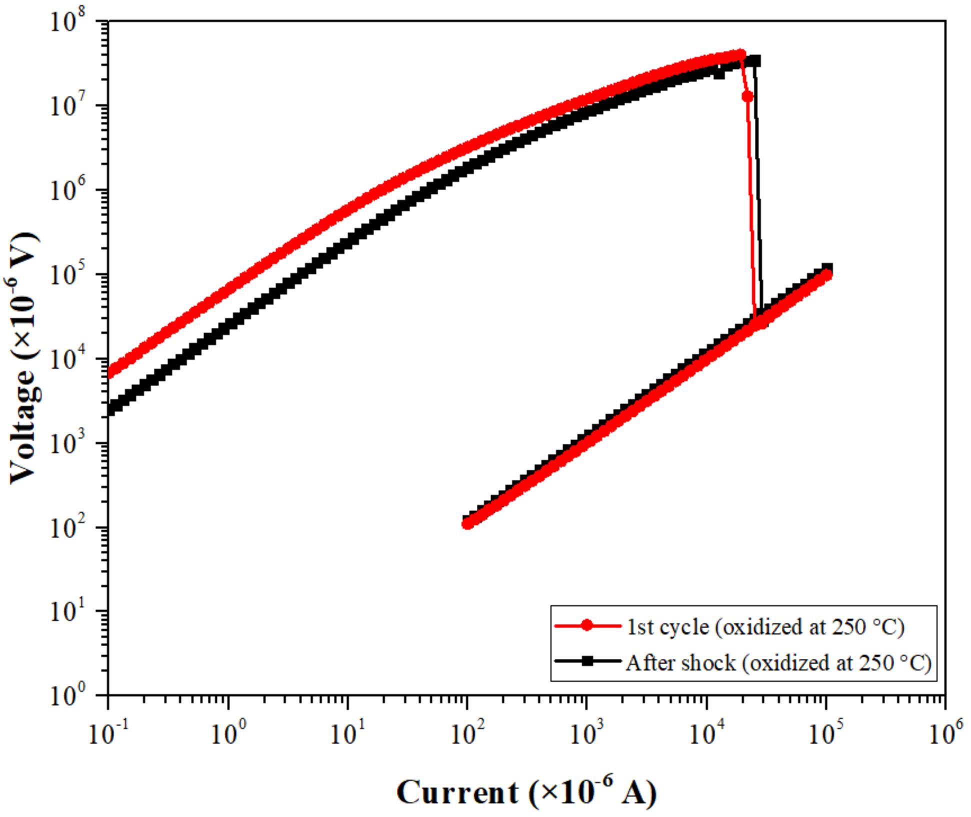

Figure S4. Typical U−I characteristics measured during current cycles up to 0.1 A for oxidized Cu powders at 250 °C before and after mechanical shock.

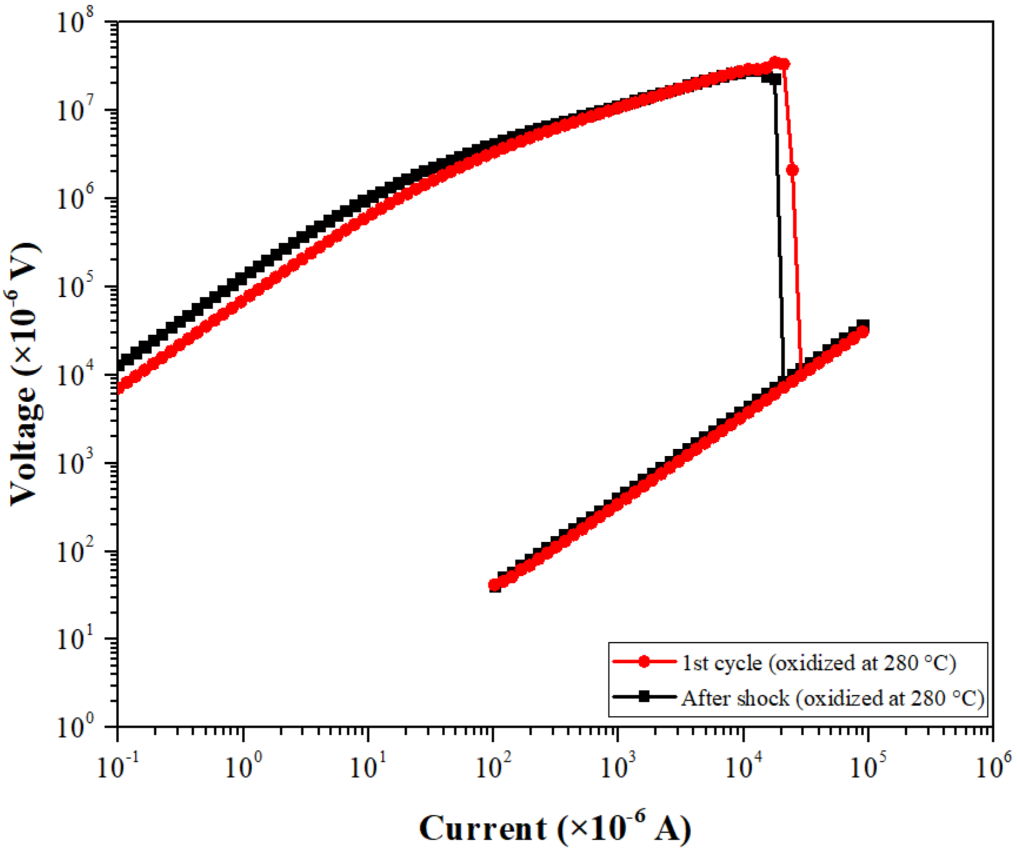

Figure S5. Typical U−I characteristics measured during current cycles up to 0.1 A for oxidized Cu powders at 280 °C before and after mechanical shock.
